# The Search for Personality–Intelligence Relations: Methodological and Conceptual Issues

**DOI:** 10.3390/jintelligence6010002

**Published:** 2018-01-02

**Authors:** Phillip L. Ackerman

**Affiliations:** School of Psychology, Georgia Institute of Technology, 654 Cherry Street, Atlanta, GA 30332, USA; plackerman@gatech.edu; Tel.: +1-404-894-5611

**Keywords:** personality, intelligence, traits

## Abstract

Prior to empirical investigation of trait level measures, it had been suggested that, on balance, well-adjusted individuals tended to have a higher level of intelligence than poorly adjusted individuals. The underlying inference was that there should be positive correlations found between personality traits associated with “adjustment” and intelligence, at least at the level of general mental abilities. Over the last several decades, empirical research has suggested that, while there are sources of common variance among personality and intellectual ability measures, the relations are more scattered and provide few general findings (other than broad assessments of neuroticism and so-called engagement traits and intellectual abilities). The status of the empirical research foundation is briefly reviewed. Conceptual and methodological issues, such as non-linear relations, typical and maximal behaviors, contextualized assessment, and missing linkages are discussed in an effort to explore personality and intelligence traits in a manner that might better reveal underlying relations between these domains.

## 1. Background

Although early philosophers argued that high intelligence and good “character” go together (e.g., Plato, see [1]), until the last century there was no empirical basis for estimating the specific relationships between personality and intelligence. In modern times, Lorge [2] first reviewed the literature on personality-intelligence relations, including roughly 200 correlations “between intelligence and some scale of personality function” (p. 277). His summary indicated that “the correlation of intelligence with measures of personality range from +0.79 to −0.49 with a median at +0.04. Half the correlations, on the basis of absolute size, range between 0.00 and 0.15 and only one fourth of them are greater than 0.30” (pp. 277–278, [2]). Some of the correlations he examined included clinical samples, while others were done with non-clinical samples, which could possibly account for some of the larger observed correlations. Fifty-seven years later, Ackerman and Heggestad [3] provided a meta-analysis of 2033 correlations among studies of non-clinical samples, between a wide variety of personality trait measures and 11 different intellectual abilities. Although many estimated true-score correlations were significantly different from zero, the correlations were mostly modest in magnitude (with the exception of measures of intellectual “engagement”, such as openness to experience, and measures of test anxiety and neuroticism, which both had larger correlations with some intellectual abilities). Unfortunately, the intervening decades between the Lorge review and the Ackerman and Heggestad meta-analysis provided relatively little in terms of theory-based predictions regarding which personality traits should be related to particular intellectual abilities. The goal of the current paper is to survey the conceptual and methodological issues that might help explain the pattern of results obtained to date, and to explore solutions that could lead to improved understanding of why, how and where these important domains may be associated with one another.

### 1.1. Why Should Personality Traits and Intellectual Abilities Be Related to One Another?

One observation into relations between intelligence and non-ability traits was offered by Thorndike [4], though without any presentation of empirical data to support the point. Thorndike wrote:

“With few or no exceptions superiority in one desirable trait implies superiority in any other. The various sorts of intelligence (with abstractions and symbols, with things and mechanisms, with people and their motives) are positively related; intelligence in general is correlated with virtue and goodwill toward men; both are correlated with skill in control of hand, eye, voice, etc.; all these are correlated with health, poise, sanity, and sensitiveness to beauty. Some of these intercorrelations are low, but they are rarely zero or negative. There is, I think, no demonstrated case of a negative correlation in all the work so far done.”.

(pp. 273–274, [4])

Notwithstanding the lack of data, it is useful to consider what Thorndike meant by suggesting that higher levels of intelligence were associated with positive aspects of personality-type traits. The array of potentially interesting personality traits is very large indeed [5], making it very difficult to decide on particular traits to examine for intelligence relations. There might be a reasonable consensus among researchers and practitioners that personality assessments indicating the absence of psychopathology would be a good place to start. Beyond clinical diagnoses, one might also suggest that “good” personality might be associated with higher levels of need for achievement or conscientiousness, well-being, openness to experience, and similar constructs; and lower levels of anxiety or neuroticism, and psychotocism. Whether the remaining “dark triad” constructs (i.e., narcissism and Machiavellianism) represent “bad” personality traits is perhaps somewhat controversial.

Cattell [6], for example, attempted to explain (post-hoc) the relations he found between personality estimates (ratings on 35 trait clusters) and measures of intellectual ability (Army Alpha test and the Graduate Record Examination). For a finding that extroversion-related traits were negatively associated with verbal intellectual ability, Cattell suggested that a person who preferred the company of books to other people would more likely end up with a greater vocabulary, and thus higher verbal ability. One potential implication of this hypothesis is that there should be an increasingly negative correlation between a measure of extroversion and verbal ability over the course of child and adolescent development—something that has yet to be clearly evaluated one way or another. While one could criticize Cattell’s post hoc explanation of this finding, it is important to point out that it is one of the relatively few hypotheses that address the *how* question: that is, specifying how personality traits could influence individual differences in intellectual abilities. As yet, there do not appear to be any salient explanations of how intellectual abilities might influence individual differences—in particular, personality traits—though the promising results of the recent work by Ziegler and his colleagues associated with the openness-fluid-crystallized intelligence model should be noted [7,8].

Other personality traits, however, are truly bipolar, such as dominance/submissiveness or agreeableness, where scores at both tails of the distribution are associated with less desirable characteristics than scores in the middle of the distribution. Traits conceptualized in this manner would necessarily be expected, from extrapolating Thorndike’s view, to evidence curvilinear relations with intelligence, in the form of an inverted-U. Still other traits may have largely ambiguous status with respect to what is “good”, such as traditionalism, depending both on the individual’s environmental constraints and an observer’s value system. Under those circumstances, stating a priori which direction of correlation should exist to support Thorndike’s suggestion would inevitably be problematic.

### 1.2. Which Traits?

Based on the reasoning above, determining which personality traits should be associated with which intellectual ability traits is a task that varies considerably in difficulty. One initial concern has to do with delineating taxonomically what the universe of traits is within each domain and, consequently, what level of specificity is needed to determine the associations between constructs within the personality and ability domains. A bottom-up approach that examines the literature for replicable findings is a good initial strategy. While this is a useful framework for assessing the status of the field, there are two main limitations of this approach. First, it is largely atheoretical when it comes to predictions about which personality traits should be associated with which intellectual ability traits, and second, it focuses on the research that has been conducted to date, leaving large gaps in knowledge about personality–intelligence relations for traits that have not been jointly investigated.

There are, however, some theories that make explicit mention of personality trait–intellectual ability trait relations. One theory is drawn from Cattell’s “investment hypothesis”. Historically, Cattell’s [9,10,11] theory of intelligence and Hebb’s [12] theory, from which Cattell’s theory was adapted), was fundamentally different from the dominant view by Spearman [13,14]). Specifically, Spearman’s approach suggested that intelligence was largely inherited and fixed, while Cattell’s approach was largely developmental. Although he defined fluid intelligence (Gf) much along the lines of Spearman’s *g*, crystallized intelligence (Gc) was hypothesized to develop as a function of the individual’s interactions with the environment, including the effects of education. From this framework, Cattell suggested that individual differences in some non-ability traits, including personality traits, influenced the direction of effort toward development of Gc. Later researchers, such as Welsh [15], focused on a construct called “intellectance”, which he defined as “the personality dimension related to performance on intellectual measures” (p. 69), but his measures of intellectance were largely a combination of both intelligence and personality items, so it is impossible to determine the relationship between intellectance and intelligence.

Although Welsh focused on overall general intelligence, I, along with my students and colleagues (e.g., [16]) have proposed a personality trait of “typical intellectual engagement” (TIE) as a reflection of the characteristics of an individual toward or away from acquiring knowledge and skills (which reflect Cattell’s Gc construct). The measure that was created to assess TIE was specifically predicted to be more highly associated with Gc intellectual abilities, which are usually assessed with measures of domain knowledge and verbal content, compared with fluid abilities, which are usually assessed with measures of abstract reasoning, short-term and working memory, and novel problem solving. Results from various investigations of this measure support the prediction that TIE is more highly related to Gc than Gf. Moreover, similar results are found for measures that correlate substantially with TIE, such as measures of openness to experience and need for cognition (e.g., see [17])—for a review and meta-analysis, see [18], for a recently articulated integrated theoretical approach, see Mussel [19].

## 2. The Current State of the Field

Since the 1980s, a common method for empirically determining the relations between constructs has been meta-analysis. That is, because individual studies of a particular question (e.g., the relationship between personality constructs and intelligence constructs) are limited in scope, because relatively too few measures are examined, or samples are decidedly non-random, or are underpowered because of small samples, and so on, the general idea is that by aggregating results across multiple studies, a researcher can go beyond simple tabulations of “for” or “against” a particular finding, and ultimately estimate the average correlation between different constructs across multiple measures and samples, typically by weighting the results of different studies by the sample size of each. Moreover, estimates of true-score correlations between traits can be accomplished when the reliabilities of the constituent measures are known, and statistical corrections for other issues, such as restriction of range of talent, can also be accomplished. Depending on the approach adopted by the researcher, one can reach a reasonable conclusion regarding what kinds of correlations between actual measures can be expected, or one might have a clue about the magnitude of the underlying theoretical relationship between two measures.

Ackerman and Heggestad [3] attempted to provide a meta-analytic summary of the relations between personality traits and intellectual ability traits. Based on their review of the literature, they found 135 studies of non-clinical samples of adolescents and adults that included at least one linear correlation between a personality assessment and an intellectual ability assessment. They classified personality traits and intellectual abilities using existing taxonomies (e.g., Eysenck, Tellegen, the Five-Factor Model, for personality; Carroll’s [20] taxonomy for abilities), which yielded a matrix of 19 personality traits and ten abilities. After statistically correcting the correlations for unreliability of the respective measures, they found estimated true score correlations between personality traits and abilities to be statistically different from zero in about half (52%) of the cells of the matrix where sufficient data existed. There were three findings from this pattern of results that are especially relevant to the current discussion, as follows: First, there were ubiquitous negative correlations between personality traits most highly associated with neuroticism, psychotocism, and test anxiety on one hand, and a variety of abilities on the other hand. Second, there were positive correlations between personality traits of intellectance, typical intellectual engagement, and openness to experience and intellectual abilities, especially those in the crystallized intelligence, fluency, and knowledge domains. Third, even though several studies reported larger correlations, estimated average true-score personality–intelligence relations were relatively modest in magnitude, rarely exceeding *r* = 0.20.

## 3. Why Substantial Relations Might Not Be Seen

There are many reasons why substantial correlations will be unlikely to be found across personality assessments and intellectual ability assessments. Four issues stand out as the most likely to result in attenuated relations. They are (1) measurement context; (2) non-linear relations; (3) bandwidth issues and Brunswik symmetry; and (4) aggregation issues. Each of these is reviewed in turn below.

### 3.1. Measurement Context

One prominent issue is the discrepancy between both the measurement context and the activation of traits for personality and intelligence. As pointed out by Cronbach [21], personality assessments mainly focus on *typical behavior*, that is, the key underlying question to be answered in a personality assessment is how the individual usually behaves across many different situations, or what would the individual prefer or like to do when there are weak influences of situational press. Intelligence assessments ever since Binet, in contrast, are typically performed under *maximal performance* conditions [22]). In other words, intelligence assessments involve situations of extremely strong environmental press 1 An individual completing a college selection battery (such as the SAT), or an intelligence test in connection with a job application, is faced with a situation that is externally controlled with constrained time limits and a rigid testing environment (with specifications for the lighting, work surface, the use of aids such as a calculator, the absence of access to a smartphone, etc.). Under such circumstances, in light of the person X situation interaction framework [24], during the testing situation, the opportunities for expression of an individual’s personal preferences for social interactions, agreeableness, and so on are extremely restricted. It is almost inconceivable that an ability testing situation would be specifically constructed in a way to instruct the examinees to respond “as they typically would behave”.

There are three approaches to address this discrepancy between typical behavior and maximal performance. One approach is to consider personality from a “maximal” perspective [25]. That is, study participants could be instructed to respond to personality assessments in terms of what they “could do” instead of how they typically behave. This approach could conceivably work reasonably well with some personality traits, such as introversion/extroversion, where the participants could be asked whether they are capable of, say, studying or reading alone for an extended period of time, or in contrast, whether they are capable of being the “life of the party”. Other traits might be more difficult to assess in a maximal context, though it would be an interesting exercise to determine whether individuals are able to “act” in a high or low neurotic, agreeable, conscientious, or open to experience fashion over an extended period of time (e.g., see [26]).

Another approach would be to determine, through objective measures or observations, whether individuals are able to act in ways that are high or low on some personality trait when given instructions to do so. Such an approach has been investigated and reported in the literature (though not in conjunction with ability correlates), and only in limited circumstances (e.g., [27,28]).

The third approach is to consider abilities in a more “typical behavior” context. This could be achieved through a variety of different observational and archival methods, such as by reviewing the fluency of casual conversations, e-mails and texts, or examining problem-solving behaviors in more naturalistic contexts than the testing situation. One could otherwise examine intellectual abilities that are more highly dependent on typical investments of time and effort (such as Gc or domain knowledge), than those that are expected to be less related to specific cognitive investments in learning and skill acquisition (i.e., Gf assessments). This was the approach taken by Goff and Ackerman [16] in their investigation of the TIE measure. The predicted results were that because Gc-type measures are largely a function of the individual’s cognitive investment, though school, work or avocational reading, such measures would be best considered as indicators of an individual’s “typical” intelligence-related activities, and as such, would be more highly associated with personality traits related to preference for intellectual activities, than Gf measures would be.

### 3.2. Non-Linear Relations

The issue of potential non-linear relationships between some personality traits and intellectual abilities is a long-standing problem in this field. Given the general orientation that higher intellectual abilities are a “good” thing, along with the ubiquity of positive manifold findings in the ability literature [29]—which means that ability measures themselves are positively correlated with one another—there is no reason to expect that higher levels of any ability would be associated with poorer functioning. However, for many bipolar personality constructs (e.g., introversion–extroversion), it is reasonable to assume that “good” personality levels are in the center of the distribution of scores, and “poor” personality levels are at the extremes, which a priori would support an inverted-U relationship between a bipolar personality trait and any number of different ability traits. Under such circumstances, a significant Pearson product moment linear correlation between a personality trait assessment and an ability assessment might not be expected across the range of potential personality trait scores. When a sample of respondents is restricted in range on the personality trait in some fashion (e.g., only assessing well-adjusted or poorly-adjusted individuals), a Pearson correlation could conceivably be positive, negative, or essentially zero. The issue becomes even more complicated when the assessment measure has ceiling or floor effects (for either the personality or ability measures), which could be expected to attenuate the correlations among personality traits and ability measures. Moreover, different personality measures will have different frequency distributions of items at the low, middle, or high ends of the trait, rendering any comparisons between different measures, in terms of non-linear regressions, fundamentally incommensurable. That is, an inflection point in a non-linear curve for one measure would likely be different from another measure. These problems make it nearly impossible to conduct meta-analyses in a manner that would capture non-linear relations between personality and ability measures (for a discussion of this issue, see [3]).

Other non-linear functions may be expected for associations between particular personality traits and intellectual abilities. It may be that the “ideal point” for a specific trait is not at one extreme or another or at the center of the distribution, but somewhere else. In fact, there is little reason to hypothesize, a priori, that the mean or median on a personality trait in the population has any special theoretical or practical significance. Such complex relationships might be expected that would be revealed by curve-fitting or other non-linear modeling. From a meta-analysis of need for achievement (nAch) measures, Spangler [30] suggested that the presence or absence of incentives for the criterion measures (e.g., grades, IQ scores) and the kind of incentives (social vs. activity incentives) may moderate the relationship between nAch measures and outcome criteria. Under these circumstances, it would not be possible to make a *general* prediction of the degree of association between a personality trait and an intellectual ability trait, but an association may nonetheless be both measurable and predictable under particular circumstances. Similarly, other sources of personality-behavior interactions in different situations, such as suggested by trait activation theory [31], would be expected to result in other kinds of non-linear relations between personality trait measures and intellectual abilities across multiple contexts. 

### 3.3. Bandwidth Issues and Brunswik Symmetry

An overarching issue in exploring the relations between personality traits and intellectual abilities is the determination whether there should be associations at the level of broad or narrow traits in both domains. There is extensive theory in both areas regarding the structure of the respective trait domains. For intellectual abilities, there is broad agreement that abilities can be represented by a hierarchy, with general intelligence (*g*) at the top of the hierarchy, broad abilities (e.g., Gf, Gc, spatial ability, fluency, math ability, perceptual speed, knowledge) at the next lower level, and a wide variety of narrower abilities at a third level (e.g., for Gc, constituent abilities include cerbal comprehension, vocabulary, reading speed, writing, etc.). This framework is often referred to as the Cattell–Horn–Carroll (CHC) theory [32]. 

For personality traits, there is substantial controversy about the structure of personality traits, and the level-of-analysis that is best suited to studying the role of personality traits in a variety of different situations. Although the five-factor model (FFM) is currently a dominant representation for capturing the main sources of variance in personality in some quarters, other approaches have their advocates and their critics (e.g., a single general personality factor [GFP], the Eysenck 3-factor model, or Tellegen’s 11-factor model—see, for example, [33]; or even differences in the structure of personality traits across cultures). Some researchers largely dispense with attempting to map out a universal structure of personality traits, in favor of examining a subset of traits (e.g., the Dark Triad—psychoticism, narcissism, and Machiavellianism, see [34], or even facets of individual traits (e.g., facets of Openness to Experience, see [35], for exploring relations with other domains.

Wittmann and Süß [36] have provided an important framework called “Brunswik symmetry” for considering how to maximize relations between predictor and criterion measures that takes account of bandwidth concerns, but also the domains in which these measures should be aligned. In their framework, maximal correlations (validity) are obtained when both the breadth of the respective measures are equivalent and when there is theoretical correspondence between the measures. For an example of this type of approach to personality-intelligence relations, see the work by Beauducel et al. [37]. Adopting this kind of approach suggests that one could consider, for example, whether there are any general personality traits (e.g., GFP) that would correspond to a general intellectual ability, or whether there are narrower personality traits that would correspond in some fashion to narrower intellectual abilities.

The argument that GFP should be related to general intelligence is reminiscent of Thorndike’s [4] statement, to the degree that GFP is weighted mainly in terms of relatively unipolar positive traits (e.g., low neuroticism, high agreeableness, openness to experience, and so on—see [38,39]). Some evidence has been obtained that a GFP measure is somewhat related to a general factor of intelligence. For example, Schermer and Vernon [40] reported correlations of approximately *r* = 0.27 between a GFP from the Personality Research Form and a general ability estimate from the Multidimensional Aptitude Battery. Still, the GFP approach remains controversial overall [41]).

In contrast, the trait of openness to experience and other related “engagement” personality traits are much narrower than the GFP. From a Brunswik symmetry perspective, one might not expect these traits to be most highly associated with broad measures of intellectual ability. In addition, as mentioned earlier, the TIE measure was specifically theorized to be more highly related to Gc-type abilities than to Gf-type abilities. The meta-analysis of such engagement measures and intellectual abilities by von Stumm and Ackerman [18] largely supported this notion, with estimated mean correlations for a variety of engagement measures and assessments of Gc-type abilities of approximately *r* = 0.30, and for TIE and measures of Gc, the correlations often exceed *r* = 0.40 in the literature [42].

### 3.4. Aggregation Issues

Although meta-analysis has become a dominant methodology for summarizing and integrating findings across studies, especially when the literature largely consists of somewhat under-powered studies, aggregation of measures and experimental contexts can have the effect of yielding attenuated effect size estimate, when there are underlying measure or contextual differences that interact with the underlying personality-intelligence relationships [43]. The typical strategy for addressing these issues is to perform moderator analyses, but such an approach is dependent on the availability of data from conditions that vary across the likely moderator variables (e.g., cultural context, age of respondents, specific measures of personality traits and intelligence traits). When there are insufficient data to perform such an analysis, a meta-analysis might be usefully supplemented by a best-evidence synthesis [44], where the researcher focuses on findings that are obtained from the “best” studies in the literature, rather than aggregating across disparate studies of lower diagnosticity (e.g., small samples, under-sampling of traits, and so on). 

## 4. Strategies for Finding Personality-Intelligence Relations

Based on the issues outlined above, several strategies are proposed that may help demonstrate more substantial relations between personality traits and intellectual abilities. These strategies pertain to (1) evaluating bipolar personality traits; (2) examining missing linkages between personality and intelligence; (3) assessing other intellectual ability criteria; (4) expanding the assessment of personality beyond self-report assessments of typical behaviors; and (5) adopting a whole-person assessment approach to include other non-ability traits. Each of these is discussed in turn below.

### 4.1. Bipolar Personality Traits

When investigating the relationship between bipolar personality traits and intellectual abilities, the first requirement should be that the personality scale in question be constructed so that it equally discriminates across the range of possible scores. Any ceiling or floor effects will likely obscure the underlying relationships. A second requirement should be that there is an a priori specification of the shape of the curve relating the personality trait to one or more ability traits. The inverted-U shape mentioned earlier would be appropriate when optimal adjustment is associated with scores in the middle of the distribution, but other curves might be theoretically justified. A third requirement is to take account of any restriction of range in the sample. This is normally a major concern across studies of intellectual abilities, especially when the sample is pre-selected on abilities, such as when dealing with college students as study participants. But, this is a little-noticed concern when examining personality traits, especially when one might expect the relationship between the underlying personality trait and the ability trait to be non-linear. Finally, one needs to take account of the fact that the inflection point of any curvilinear relationship is likely to be dependent on the particular personality trait measure. Few, if any, credible theories in psychology could reasonably predict that any particular sample median value represents a likely inflection point, despite the presence of some popular typologies of personality traits.

### 4.2. Missing Linkages

Two additional findings from the meta-analysis and review of personality-intelligence-interest relations conducted by Ackerman and Heggestad [3] are relevant to the current discussion, not because they illustrated linkages between personality traits and intellectual abilities, but because the indicated missing linkages. Of the four broad trait complexes (constellations of personality–intelligence–interest traits that had overlap), two of the trait complexes stood out as indicating something was missing. The science/math trait complex, which included math and spatial abilities, had no overlapping personality traits, and the social trait complex, which included extroversion and well-being personality traits, included no abilities. The obvious question is whether there exist personality traits, either unidentified or not studied in this context, that would be significantly associated with science/math abilities. One relevant investigation of related issues was a study conducted by Toker and Ackerman [45], where an attempt was made to find self-concept and interest measures that were associated with enrollment/engagement in science, technology, and math majors in university study. Results were supportive of the notion that there are self-perceptions that are related to this domain, but these traits are not univocally associated with existing personality constructs.

With respect to the social trait complex, the clear missing link is some intellectual ability that could be identified as “social intelligence”—that is, the ability to competently act in interpersonal situations. Early in the history of modern intelligence research, several investigators attempted to construct reliable and valid measures of social intelligence [46], but they largely failed to develop assessments that were not otherwise subsumed under existing ability constructs. It may be that an entirely new method of assessing social intelligence is needed—perhaps one that is contextualized within the individual’s sphere of contacts. That is, instead of presenting examinees with novel or arbitrary scenarios, the key to social intelligence may be the individual’s familiarity with a set of other individuals, and knowledge about how best to interact with those individuals. Such assessments may be difficult to perform, but they could potentially have both higher discriminant validity with existing ability measures and higher convergent validity with personality constructs of extroversion and well-being, and with external criteria, such as job performance in group or team situations.

### 4.3. Other Ability Criteria

If the goal is to find relations between personality traits as “typical” behaviors and intellectual abilities, then it seems clear that one should move beyond the assessment of intelligence in the traditional maximal performance situations. As discussed earlier, examining aspects of Gc which involve investment of cognitive resources over extended periods of time and which are most likely to reflect “typical“ engagement of the individual represents one promising area for finding substantial personality–intelligence relations. School grades, for example, represent a combination of Gc-type abilities and others (when grades are determined both by assignments over the course of a school term, but also in-class examinations). In a meta-analysis, Poropat [47] has shown that there are several sources of shared variance between personality trait measures and assessments of academic performance. However, concentrating on Gc or school grades leaves out a good number of other sources of intellectual performance. Naturalistic problem-solving of math and spatial tasks, critical thinking, game-playing performance, learning outside of the classroom context, and other potential sources exist for the examination of other intellectual abilities as a typical behavior. These relatively narrow intellectual domains probably would be most highly related to similarly narrow personality traits, in consideration of Brunswik symmetry.

### 4.4. Beyond Self-Report Personality Assessments of Typical Behaviors

Another approach to investigating personality traits beyond typical behaviors that may be fruitful is to contextualize the assessments. A few investigations have suggested that it is potentially useful to consider that, within individuals, expressions of some personality traits (e.g., conscientiousness, extroversion) may be different depending on the context. An individual might be neat, tidy, and punctual, or even gregarious or affiliative when at home or meeting with fiends, but not so much at work or at school [48,49]. Rather than focusing on the general trait, which is assumed to average across many different situational contexts, it may be that when individuals are asked to report their typical behaviors in contexts that require intellectual activities, relationships between the personality traits and intellectual abilities are accentuated.

Similarly, efforts to expand assessments of both personality beyond self-report and intellectual abilities beyond the one-on-one or group testing methods might be expected to reveal other sources of personality–intelligence relations. The use of assessment centers [50] or field experiments, along the lines the Robber’s Cave experiment [51], where interventions are used in a naturalistic setting with groups, might be used to jointly identify personality trait expression and level of intellectual effectiveness in a variety of different ways. Independent observers could score these different characteristics in a more in-depth stream of behavior than is otherwise captured by standardized testing situations. (For a discussion of traditional versus behavioral personality assessment, see [52]). For a similar discussion regarding intelligence assessment, see [53].) The underlying theme here is to eliminate the strong situational press that is present in traditional assessments of both personality traits and intellectual abilities.

### 4.5. Expand the Domains—Trait Complexes

One approach to improving understanding of the relations between personality and intelligence is to take a broader conceptualization of the person than the traditional list of personality traits and intellectual abilities. Interests, for example, are often considered to be tightly integrated with personality, but until the last couple of decades, investigators have largely ignored their associations [54]. However, some interests (e.g., investigative, artistic) have substantial associations with intellectual abilities (math and spatial for investigative interests, verbal and fluency abilities with artistic interests), and other interests have substantial associations with personality traits (e.g., social interests and introversion/extroversion). Other traits may provide important linkages with both personality traits and abilities, such as attitudes, motivational traits, self-concept, self-efficacy, life goals, and so on. By expanding measurement to a “whole-person assessment” [55] including these various domains, it may be possible to derive a more integrated framework to understand how personality traits interact with other traits to influence individual differences in intellectual abilities, especially in a longitudinal design, where it may be possible to examine how the traits interact dynamically during child, adolescent and adult development.

As a partial attempt towards whole-person assessment, several investigators have focused on examining trait complexes, representing commonality among personality trait measures, intellectual ability measures, along with interests, self-concept, and motivational traits. Snow [56] was the first to suggest that such complexes might be facilitative or impeding of learning. Our investigations have focused on whether these trait complexes are related to individual differences in both existing knowledge and the acquisition of new knowledge. From this perspective, the “intellectual/cultural” trait complex (which includes Gc abilities and engagement personality traits, along with Artistic and Investigative interests, and self-estimates of verbal abilities), has been found to be positively related to individual differences in intellectual domain knowledge among late adolescents and adults, while the social trait complex (which includes extroversion-related personality variables and social and enterprising interests) is generally negatively related to these same outcome variables [42,57,58]. These results are consistent with the notion that personality traits are less influential as independent predictors of learning and knowledge, but rather that personality traits, in conjunction with other trait families, jointly influence the outcomes. That is, just having a high level of openness to experience may not influence the acquisition of foreign language skills. However, when coupled with specific interest in, and motivation for, acquiring such skills along with high levels of the abilities requisite for knowledge acquisition, higher levels of openness to experience may contribute to a successful learning outcome. The investigation of higher-order interactions involving multiple trait families represents a significant challenge for the design of empirical studies, because such approaches are typically statistically under-powered [59].

## 5. Conclusions

Over the course of nearly eight decades since Thorndike observed, without any specific data, that desirable traits tend to be positively correlated with one another, the corpus of empirical research has found that there is some support for this assertion. With the exception of neuroticism-related traits and intellectual engagement traits, the relations between personality measures and intellectual ability measures are significant, but relatively modest in magnitude. The lack of substantial correlations between other personality trait measures and intellectual ability measures may be more likely due to the lack of appropriate methods for assessing personality–intelligence relations, and partly due to the lack of specific predictions that are based in mapping personality theories to activities that are dependent on intellectual abilities.

In this paper, I have outlined the limitations of traditional approaches to assessing personality–intelligence relations, such as restricting investigations to examining linear correlations. I suggest that broadening the search for personality–intelligence relations by systematically examining non-linear relationships, especially for bipolar personality traits, specifically matching the breadth and specificity of both predictor and criterion assessments, looking for missing linkages between these domains, and taking a whole-person assessment perspective may prove fruitful avenues of future research. It may be a function of unrepentant optimism, but the success of a small number of investigations that have taken one or more of these approaches appears to point to a way forward that might revitalize the field. Such approaches might also bring a more integrative perspective to understanding how various personal characteristics interact to yield individual differences in acquired intellectual abilities across the lifespan.

## Footnotes

1 A similar point was made in passing by Cattell [23]. “... there has been a tacit or unconscious conspiracy to maintain certain influences constant when giving tests, without mentioning—often within realizing—that such artificial conditions have been set up. We correlate ability tests under conditions of quiet, of concentration, of common intention to one’s best. We correlate emotional responses in, say, nursery school children, observed under conditions in which cognitive abilities are not required in order to manifest emotion.” (p. 133).
